# Soluble receptor for advanced glycation end products (sRAGE) as a biomarker of COVID-19 disease severity and indicator of the need for mechanical ventilation, ARDS and mortality

**DOI:** 10.1186/s13613-021-00836-2

**Published:** 2021-03-22

**Authors:** Adeline Lim, Aleksandar Radujkovic, Markus A. Weigand, Uta Merle

**Affiliations:** 1 Department of Internal Medicine IV, University Hospital Heidelberg, Im Neuenheimer Feld 410, 69120 Heidelberg, Germany; 2 Department of Internal Medicine V, University Hospital Heidelberg, Im Neuenheimer Feld 410, 69120 Heidelberg, Germany; 3 Department of Anesthesiology, University Hospital Heidelberg, Im Neuenheimer Feld 410, 69120 Heidelberg, Germany

**Keywords:** Soluble RAGE, RAGE, COVID-19, Biomarker, Disease severity, ARDS, Prediction, Pneumonia

## Abstract

**Background:**

COVID-19 pneumonia and subsequent respiratory failure is causing an immense strain on intensive care units globally. Early prediction of severe disease enables clinicians to avoid acute respiratory distress syndrome (ARDS) development and improve management of critically ill patients. The soluble receptor of advanced glycation endproducts (sRAGE) is a biomarker shown to predict ARDS. Although sRAGE level varies depending on the type of disease, there is limited information available on changes in sRAGE levels in COVID-19. Therefore, sRAGE was measured in COVID-19 patients to determine sRAGE level variation in COVID-19 severity and to examine its ability to predict the need for mechanical ventilation (MV) and mortality in COVID-19.

**Methods:**

In this single-centre observational cohort study in Germany, serum sRAGE during acute COVID-19, 20 weeks after the start of COVID-19 symptoms, as well as in control groups of non-COVID-19 pneumonia patients and healthy controls were measured using ELISA. The primary endpoint was severe disease (high-flow nasal oxygen therapy (HFNO)/MV and need of organ support). The secondary endpoints were respiratory failure with need of MV and 30-day mortality. The area under the curve (AUC), cut-off based on Youden’s index and odds ratio with 95% CI for sRAGE were calculated with regard to prediction of MV need and mortality.

**Results:**

Serum sRAGE in 164 COVID-19 patients, 101 matched COVID-19 convalescent patients, 23 non-COVID-19 pneumonia patients and 15 healthy volunteers were measured. sRAGE level increased with COVID-19 severity, need for oxygen therapy, HFNO/MV, ARDS severity, need of dialysis and catecholamine support, 30-day mortality, sequential organ failure assessment (SOFA) and quick SOFA (qSOFA) score. sRAGE was found to be a good predictor of MV need in COVID-19 inpatients and mortality with an AUC of 0.871 (0.770–0.973) and 0.903 (0.817–0.990), respectively. When adjusted for male gender, age, comorbidity and SOFA score ≥ 3, sRAGE was independently associated with risk of need for HFNO/MV. When adjusted for SOFA score ≥ 3, sRAGE was independently associated with risk of need for MV.

**Conclusions:**

Serum sRAGE concentrations are elevated in COVID-19 patients as disease severity increases. sRAGE should be considered as a biomarker for predicting the need for MV and mortality in COVID-19.

**Supplementary Information:**

The online version contains supplementary material available at 10.1186/s13613-021-00836-2.

## Background

The current COVID-19 global pandemic is caused by the virus SARS-CoV-2 and has a case-fatality ratio ranging from 1.3 to 9.8% as of 14th Nov 2020 [1]. In COVID-19 patients with severe disease progression, a hyperinflammatory status, severe endothelial damage, thromboembolic diseases, micro- and macroangiopathy, capillary leakage resulting in acute respiratory distress syndrome (ARDS) requiring mechanical ventilation, and multiorgan failure can be observed [2]. Among the many organ systems affected, COVID-19 pneumonia is the most common and causes the greatest need for intensive care support [3].

There are a number of biomarkers that indicate severe disease progression in COVID-19. The aim of this study was, therefore, to identify a reliable biomarker that can accurately reflect COVID-19 disease severity to enable early detection of patients needing MV and allow close monitoring, early prevention and early treatment to be carried out.

Knowledge regarding the pathophysiology of SARS-CoV-2, which allows such therapeutic intervention, is limited. The receptor for advanced glycation end products (RAGE) facilitates inflammation, the immune response to infection and subsequent endothelial damage [4, 5] and is expressed most prominently on alveolar epithelial cells in the lungs [6–8]. The soluble RAGE (sRAGE) in serum has the extracellular domain of RAGE but lacks the transmembrane and intracytoplasmic domains [9]. sRAGE binds to RAGE ligands without activating the RAGE-mediated signaling pathway, thereby acting as a competitive inhibitor of RAGE. There are many isoforms of sRAGE [9, 10] and their measurement is receiving increasing attention as reporters of immune system function.

sRAGE level in blood correlates with high levels of ongoing bacterial infection, inflammatory diseases [11], indicates lung epithelial injury [12], and is known as a predictor of the development of ARDS in non-COVID-19 patients [13]. Furthermore, it has been hypothesized that the RAGE pathway plays a central role in the pathogenesis of COVID-19 [14, 15]. For these reasons, we hypothesised that serum sRAGE may indicate COVID-19 disease severity, defined as the highest level reached on the WHO clinical progression scale [16] in each individual patient.

sRAGE levels in COVID-19 patients were, therefore, measured to verify the reliability of sRAGE in predicting the need of mechanical ventilation (MV) and mortality in COVID-19 patients.

## Methods

### Study design and patient selection

Consecutive symptomatic SARS-CoV-2-positive patients treated at the University Hospital of Heidelberg were screened for this prospective non-interventional register with biobanking study, that was approved by the Ethics Committee of the Medical Faculty of Heidelberg University Hospital (number S-148/2020) and in accordance to the Declaration of Helsinki [17]. All patients with laboratory-confirmed SARS-CoV-2 infection as well as patients suspected of SARS-CoV-2 infection aged 18 years or older treated as out- or inpatients between 18.03.2020 and 02.10.2020 were considered for inclusion in this study. Written informed consent was obtained for all patients included in the study. Patient data and serum samples were collected prospectively. Additionally, patients who were seen as post-COVID-19 patients in follow-up and who gave their informed consent were included in a follow-up study, that was approved by the Ethics Committee of the Medical Faculty of Heidelberg University Hospital (number S-546/2020) and in accordance with the Declaration of Helsinki [17]. In this follow-up study, patient data and serum samples were collected prospectively. Previously not collected additionally needed patient data were assessed in retrospect by review of the records.

The diagnosis of COVID-19 was based on a positive detection of viral genome in nasopharyngeal swabs or airway surface liquid using reverse-transcriptase quantitative polymerase chain reaction (RT-qPCR) [18]. CT imaging of the lungs was used as a supportive diagnostic criterion for ARDS according to the discretion of the attending physician. In general, HFNO was initiated when 4 L/min of oxygen was insufficient to reach a peripheral oxygen saturation of 93%, whereas MV was initiated when the Horowitz index was < 100. However, patients were managed also according to the discretion of the attending physician, taking into account factors not reflected in our data, such as subjective dyspnoea and breathing mechanics.

The COVID-19 patients were divided into four cohorts: mild disease (cohort A), moderate disease (cohort B), severe disease (cohort C) and convalescent (cohort D). Cohorts A, B and C were divided according to the WHO clinical progression scale of COVID-19 [16] according to the stage of their most severe disease progression. The cohort with mild disease (cohort A) consisted of outpatients who were visited by medically trained staff at their homes after reporting symptoms such as light dyspnoea or continuously high fever (≥ 38.3 °C). The cohort with moderate disease (cohort B) consisted of inpatients with neurological symptoms or dyspnoea requiring no or low flow oxygen therapy using nasal prongs treated at the non-intensive care ward. The cohort with severe disease (cohort C) consisted of inpatients treated at the intermediate care and intensive care unit requiring high-flow nasal oxygen therapy or endotracheal intubation and mechanical ventilation. Non-invasive ventilation was not used at our center for COVID-19 patients. In addition to acutely ill patients, a cohort of convalescent patients 20 weeks after initial onset of symptoms was analysed (cohort D). Of all patients contacted for follow-up, only those who were in cohort A, B or C were included in cohort D. The non-COVID-19 pneumonia control group consisted of patients admitted under the suspicion of having COVID-19 but who tested negatively by PCR for SARS-CoV-2 in nasopharyngeal swabs. The healthy controls were volunteers with no previous medical history.

sRAGE was measured in the earliest blood sample available. Other exclusion criteria for this study were patients younger than 18 years old, patients who did not consent to this study and patients from whom blood samples were not available within 7 days after admission or after the first house call.

The demographic characteristics, medical history, clinical and laboratory data were collected during COVID-19 infection and during follow-up. The primary outcome was severe disease (defined as the need of HFNO/MV, need for catecholamine therapy or need for dialysis). The secondary outcomes were respiratory failure with need of MV and 30-day mortality. Clinical variables measured were SOFA score, qSOFA score [19] and the Horowitz Index (PaO_2_:FiO_2_) [20].

sRAGE levels in serum were measured using an enzyme-linked immunosorbent assay (ELISA) kit from R&D Systems (Minneapolis, MN, USA). Patient sera were obtained by centrifuging whole blood for 15 min at 1800 × *g*. The pipetted sera were stored at − 80 °C until shortly before ELISA was carried out. The accuracy of the ELISAs carried out were controlled using Quantikine® ELISA Kit Controls, Control Set 832 from R&D Systems (Minneapolis, MN, USA). For sera with RAGE concentrations that were too high to be measured, an initial dilution of 1:3 was carried out, with one part serum and two parts Calibrator Diluent RD6-10. Subsequently, when the RAGE concentrations were still too high under the 1:3 dilution, a dilution of 1:5 was carried out, with one part serum and four parts Calibrator Diluent RD6-10. For sera with RAGE concentrations that were still too high under the 1:5 dilution, a maximum sRAGE concentration of 25,000 pg/mL (5000 pg/mL × 5) was assumed. There were no sera with RAGE concentrations below the lower ELISA detection limit. The means of sRAGE duplicates were reported.

### Statistical analysis

The sample size was not predefined, as all patients with written consent and met the inclusion criteria were included. To choose the appropriate statistical tests, descriptive statistics was used initially to determine the normality of data distribution across each level of comparison and homogeneity of variance of data. sRAGE levels showed a left skewed distribution and were therefore subject to natural log (Ln) transformation for analysis. Continuous variables are reported as median with interquartile range. Categorical variables are expressed as numbers with percentage. Parametric tests were preferred if the assumptions were fulfilled to increase the power, otherwise non-parametric tests were applied for analysis. Welch’s *t* test and Mann–Whitney *U* test were used as appropriate to determine the relationship between a continuous and categorical variable with two levels. Spearman’s correlation was used as appropriate to determine the correlation between two continuous variables. Brown–Forsythe ANOVA and Kruskal–Wallis tests were used as appropriate to compare means or rank of continuous variable across an independent variable with more than two levels. Where homogeneity of variance was not assumed, Dunnett’s T3 post hoc test was used. For the Brown–Forsythe ANOVA comparing sRAGE levels and ARDS severity, there was only one patient with mild ARDS and this was, therefore, disregarded, as it was not possible to perform this test for this group of mild ARDS. When Kruskal–Wallis test was used, significance values have been adjusted by the Bonferroni correction for multiple tests to avoid Type-I error. Wilcoxon matched-pairs signed rank test was used to compare sRAGE during acute disease and during convalescence. The effect sizes were reported as Cohen’s d or η^2^ as appropriate. Missing data and loss to follow-up were excluded pairwise in all analyses. Area under the curve (AUC) derived from the receiver operating characteristic (ROC) curved was used to determine the ability of different variables to predict analysed outcomes [21]. AUC was reported as AUC (95%CI). The thresholds were derived based on clinical relevance and Youden’s index [22]. Univariate binary logistic regression was used to determine the odds ratio (OR) with 95% confidence intervals (CI) for predicting the need for HFNO therapy or mechanical ventilation. Multivariate binary logistic regression was used to analyse the need for HFNO/MV and MV. The Hosmer–Lemeshow goodness-of-fit test was used to evaluate the adequacy of the regression model to describe the data [23]. The known risk factors for severe disease progression (male gender, age, comorbidity and SOFA score ≥ 3 [24–26]) were fitted into the model for analysing the need for HFNO/MV. Due to limited number of events (*N* = 19), only one of the known risk factors for severe disease progression with the largest effect size in the univariate binary logistic regression was fitted into the model for predicting the need for MV. Kaplan–Meier estimation [27] was used to estimate survival, using the threshold derived from Youden’s index. The endpoint here was death of any cause. Survival was calculated from date of first sRAGE measurement up to 30 days or death of any cause. Patients who were alive were censored at 30 days. Univariate COX regression was used to determine the hazard ratio of sRAGE predicting 30-day mortality. 2-way ANOVA was used to determine the effect of two categorical variables on a continuous variable. All tests were two-sided and *p* ≤ 0.05 was considered significant. All data were analysed using IBM SPSS Statistics Version 27, Graphpad Prism 9 and Microsoft Excel.

## Results

**Fig. 1:**
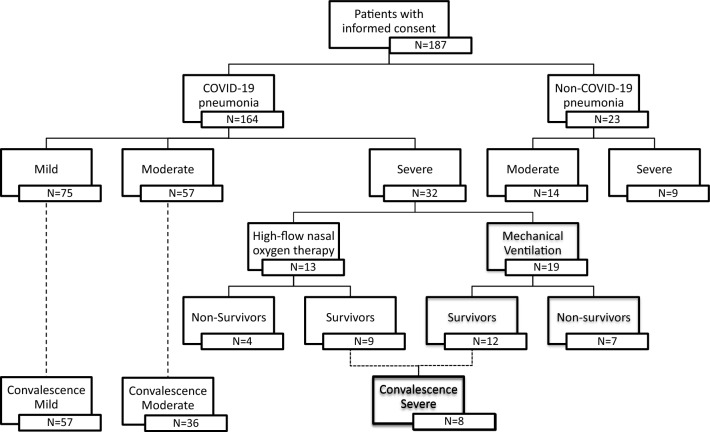
Flowchart of cohort division

187 patients with COVID-19 or suspicion of COVID-19 treated at the University hospital of Heidelberg were included in this study. Nine patients who were initially treated in other hospitals or departments were excluded from the study, as there were no blood samples available during the first 7 days of admission. There was a total of 164 COVID-19 patients, 23 patients with non-COVID pneumonia as well as 15 healthy volunteers included in this study. Out of the 164 COVID-19 patients, 101 patients came for follow-up during convalescence. The patients were further categorized as shown in Fig. 1. Table 1 shows the baseline characteristics of these cohorts. The baseline characteristics of patients with severe COVID-19 with the need of HFNO and MV were further distinguished (Additional file 1).

**Table 1:** Basic characteristics of cohorts

	COVID-19 Pneumonia (*n* = 164)			Non-COVID-19 Pneumonia (*n* = 23)		COVID-19 Convalescent	Healthy Controls
	Disease severity			Disease severity			
	mild (*n* = 75)	moderate (*n* = 57)	severe (*n* = 32)	moderate (*n* = 14)	severe (*n* = 9)	*n* = 101	*n* = 15
Gender							
Male	32 (42.7%)	31 (54.4%)	26 (81.3%)	10 (71.4%)	6 (66.7%)	48 (47.5%)	6 (40%)
Female	43 (57.3%)	26 (45.6%)	6 (18.8%)	4 (28.6%)	3 (33.3%)	53 (52.5%)	9 (60%)
Age	57 (53–60)	57 (55–64)	69 (64–77)	53 (45–69)	68 (63–81)	60 (58–62)	36 (34–52)
BMI	25.8 (24.9–26.6)	26.9 (25.3–29.4)	26.0 (24.8–26.9)	24.9 (23.1–31.6)	28.4 (26.4–31.2)	26.4 (25.7–27.4)	22.5 (20.9–25.3)
A.Hypertension	20 (26.7%)	24 (42.1%)	23 (71.9%)	9 (64.3%)	7 (77.8%)	38 (37.6%)	0
CAD	3 (4.0%)	1 (1.8%)	10 (31.3%)	3 (50%)		4 (4%)	0
Diabetes mellitus	8 (10.7%)	7 (12.3%)	8 (25.0%)	2 (14.3%)	2 (22.2%)	11 (10.9%)	0
Hyperlipidemia	14 (18.7%)	11 (19.3%)	12 (37.5%)	4 (28.6%)	4 (44.4%)	16 (15.8%)	0
Renal insufficiency	1 (1.3%)	5 (8.8%)	8 (25.0%)	6 (42.9%)	5 (55.6%)	4 (4%)	0
COPD	1 (1.3%)	4 (7.0%)	4 (12.5%)	0 (0%)	1 (11.1%)	3 (3.0%)	0
Inflammatory diseases	11 (14.7%)	9 (15.8%)	2 (6.3%)	8 (57.1%)	1 (11.1%)	16 (15.8%)	0
Malignancy	10 (13.3%)	7 (12.3%)	5 (15.6%)	0 (0%)	1 (11.1%)	13 (12.9%)	0
ACE-I	6 (8.0%)	8 (14.0%)	12 (37.5%)	4 (28.6%)	3 (33.3%)	11 (10.9%)	0
ARB	7 (9.3%)	9 (15.8%)	6 (18.8%)	2 (14.3%)	3 (33.3%)	15 (14.9%)	0
Cortisone	2 (2.7%)	5 (8.8%)	6 (18.8%)	1 (7.1%)	2 (22.2%)	5 (5.0%)	0
Statins	10 (3.3%)	11 (19.3%)	11 (34.4%)	4 (28.6%)	3 (33.3%)	16 (15.8%)	0
ASS/Clopidogrel	7 (9.3%)	6 (10.5%)	9 (28.1%)	2 (14.3%)	3 (33.3%)	8 (7.9%)	0
PaO_2_:FiO_2_		363 (329–410)	138 (106–179)	349 (307–400)	137 (118–232)		
30-day mortality	0 (0%)	0 (0%)	11 (34.4%)	0 (0%)	3 (33.3%)		
Dialysis	0 (0%)	0 (0%)	8 (25.0%)	2 (14.3%)	3 (33.3%)		
Catecholamine	0 (0%)	0 (0%)	13 (40.6%)	0 (0%)	5 (55.6%)		
SOFA	0	1 (1–2)	5 (4–7)	2 (1–6)	6 (4–13)		
Maximum SOFA	0	1 (1–2)	10 (5–13)	2 (1–6)	11 (6–14)		
qSOFA							
0	75 (100%)	46 (80.7%)	9 (28.1%)	11 (78.6%)	1 (11.1%)		
1	0 (0%)	9 (15.8%)	17(53.1%)	3(21.4%)	4 (44.4%)		
2	0 (0%)	2 (3.5%)	6 (18.8%)	0 (0%)	2 (22.2%)		
3	0 (0%)	0 (0%)	0 (0%)	0 (0%)	2 (22.2%)		

Table shows the demographic characteristics, medical history and clinical data of cohorts with COVID-19 pneumonia, non-COVID-19 pneumonia, 20 weeks convalescent and healthy controls. Nominal and ordinal variables are reported as count (percentage in cohort), continuous variables are reported as median (IQR). *BMI* body mass index, *A. Hypertension* arterial hypertension, *CAD* coronary artery disease, *COPD* chronic obstructive pulmonary disease, *ACE-I* angiotensin converting enzyme inhibitor, *ARB* angiotensin II receptor blocker, *SOFA* sequential organ failure assessment score, *qSOFA* quick sequential organ failure assessment score

### sRAGE increased in severe disease and correlated with disease severity

**Fig. 2:**
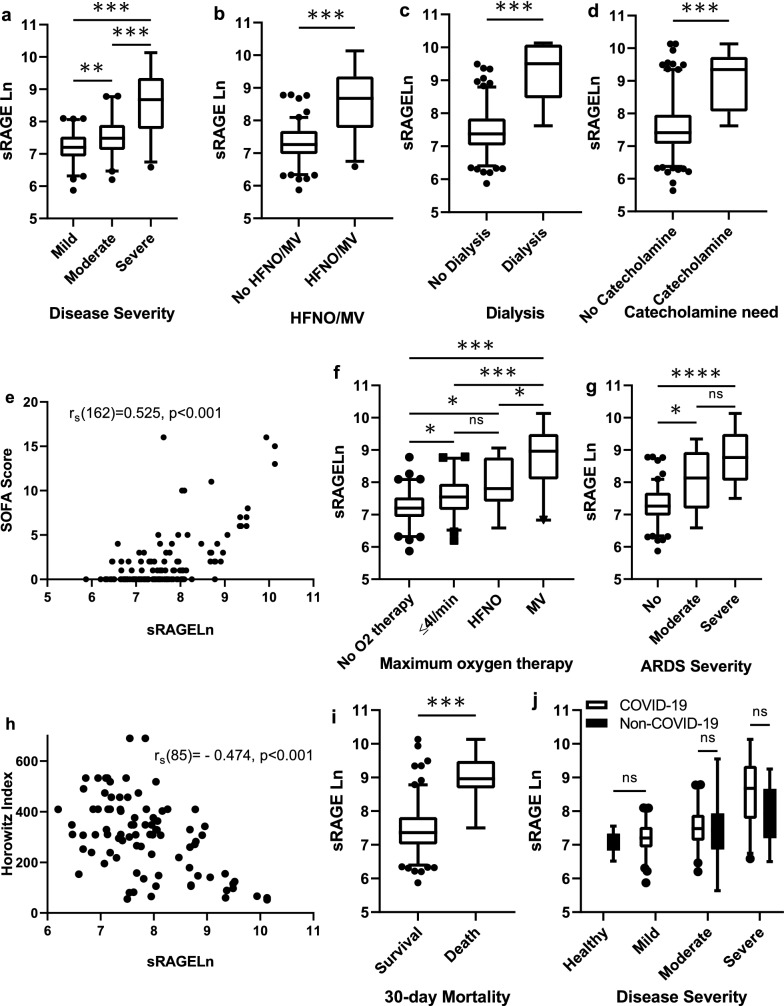
Variation of sRAGE levels with regard to disease severity. sRAGE values were subject to natural log transformation (sRAGE Ln) due to highly skewed distribution. The effect of disease severity on sRAGE Ln level for the mild, moderate and severe cohorts was significant [F*(2, 64.21) = 36.40, *p* < 0.001], sRAGE Ln increases with disease severity (**a**). sRAGE Ln was significantly higher in patients needing HFNO/MV [*t*(36.22) = − 6.81, *p* < 0.001] (**b**). sRAGE Ln was significantly higher in patients needing dialysis [U(*N*_dialysis_ = 8, *N*_no dialysis_ = 156) = 1171.00, *z* = 4.18, *p* < 0.001] (**c**). sRAGE Ln was significantly higher in patients who needed catecholamine therapy [U(*N*_catecholamine_ = 13, *N*_no catecholamine_ = 151) = 1817.00, *z* = 5.09, *p* < 0.001] (**d**). There was a significant strong positive correlation between sRAGE and SOFA score [*r*_s_ (162) = − 0.525, *p* < 0.001] (**e**). The effect of maximum oxygen and ventilation therapy required on sRAGE Ln for patients with no, ≤ 4L/min and HFNO as well as MV was significant [F*(3, 47.03) = 27.24, *p* < 0.001] (**f**). The effect of ARDS severity on sRAGE Ln for patients with no, moderate and severe ARDS was significant [F*(2, 29.69) = 26.53, *p* < 0.0001], sRAGE Ln increases with ARDS severity (**g**). There was a significant, moderately strong negative correlation between sRAGE and the Horowitz Index [*r*_s_ (85) = − 0.474, *p* < 0.001] (**h**). sRAGE Ln was significantly higher in patients who died [U(*N*_death_ = 11, *N*_survival_ = 153) = 1520.50, *z* = 4.46, *p* < 0.001] (**i**). There was no statistically significant interaction between the effects of type of pneumonia and disease severity on sRAGE Ln. Disease severity has a significant effect on sRAGE Ln but not the type of pneumonia (**j**). sRAGE Ln: natural log transformed sRAGE; high-flow nasal oxygen or mechanical ventilation; MV: mechanical ventilation

The median number of days between sRAGE measurement and the need for HFNO/MV was 0 (0–1) days. sRAGE levels correlated to disease severity, *p* < 0.001, *η*^2^ = 0.377 (Fig. 2a). sRAGE level was not only significantly higher in patients needing HFNO/MV, *p* < 0.001, *d* = 0.648 (Fig. 2b), but also increased in patients with need of hemodialysis, *p* < 0.001, *η*^2^ = 0.106 (Fig. 2c) and need of catecholamine therapy, *p* < 0.001, *η*^2^ = 0.158 (Fig. 2d). Investigating other aspects of disease severity as measured by the SOFA score, there was a significant strong positive correlation between sRAGE level and SOFA score, *r*_s_ (162) = 0.525, *p* < 0.001 (Fig. 2e). sRAGE level increases significantly as qSOFA score increases, *p* < 0.001 (Additional file 2a). sRAGE was also found to be moderately positively correlated to duration of hospitalization, *r*_s_ (89) = 0.375, *p* < 0.001 (Additional file 2b). sRAGE was significantly higher in patients with elevated D-Dimer, *p* < 0.001, *η*^2^ = 0.210 (Additional file 2c). sRAGE levels were 38% lower in the same patients during convalescence compared to during acute disease (Additional file 2d).

sRAGE distinguished HFNO/MV need in COVID-19 with an AUC of 0.853 (0.765–0.940) with 78.1% sensitivity and 81.1% specificity when set at a threshold of 2348 pg/mL. Table 2 shows odds ratios of the need for HFNO/MV based on univariate binary logistic analyses. The variable with the largest effect size was the SOFA score. Adjusting for male gender, age, comorbidity and SOFA Score ≥ 3, sRAGE Ln was associated with need for HFNO/MV (Table 3). The model was able to distinguish the need of HFNO/MV with 97% sensitivity and 90.3% specificity. Adding sRAGE to the model improved the accuracy of distinguishing the need of HFNO/MV from 95.1 to 95.7%.

**Table 2:** Odds ratio of HFNO/MV in univariate binary logistic regression analysis

	OR (95%CI), *p* value	Nagelkerke *R* Square	N/164
Gender	4.746 (1.833–12.286), = 0.001	.117	164
Age	1.077 (1.040–1.115), < 0.001	.206	164
BMI	1.012 (0.991–1.034), 0.272	.026	152
A.Hypertension	5.111 (2.182–11.974), < 0.001	.146	164
CAD	14.545 (4.190–50.497), < 0.001	.184	164
Diabetes Mellitus	2.600 (0.992–6.817), = 0.052	.034	164
Hyperlipidemia	2.568 (1.111–5.935), = 0.027	.045	164
Atherosclerosis	15.756 (5.623–44.149), < 0.001	.269	164
Renal insufficiency	7.000 (2.228–21.997), = 0.001	.102	164
COPD	3.629 (0.916–14.380), 0.067	.030	164
Inflammatory disease	0.373 (0.083–1.687), = 0.200	.020	164
Malignancy	1.253 (0.425–3.695), = 0.683	.013	164
ACE-I	5.057 (2.046–12.502), < 0.001	.111	164
ARB	1.673(0.597–4.687), 0.327	.009	164
Cortisone	4.121 (1.280–13.270), 0.018	.050	164
Statins	2.769 (1.165–6.581), 0.021	.048	164
ASS/Clopidogrel	3.582 (1.371–9.355), 0.009	.060	164
Horowitz index	0.975 (0.964–0.985), < 0.001	.747	87
SOFA	13.947 (4.162–46.736), < 0.001	.855	164
Maximum SOFA	27.437 (3.411–220.674), = 0.002	.934	164
qSOFA	13.217 (5.508–31.715), < 0.001	.340	164
Lactate	1.238 (1.096–1.399), = 0.001	.351	81
sRAGE Ln	9.509 (4.343–20.819), < 0.001	.472	164
Creatinine	10.894 (2.685–44.202), = 0.001	.306	163
Blood urea nitrogen	1.077 (1.038–1.116), < 0.001	.347	162
Troponin-T	1.201 (1.099–1.312), < 0.001	.569	100
Lactate dehydrogenase	1.009 (1.005–1.012), < 0.001	.369	162
AST	1.013 (1.004–1.022), = 0.005	.188	94
Bilirubin	6.996 (1.573–31.116), = 0.011	.180	89
Iron	0.708 (0.582–0.861), = 0.001	.241	157
Transferrin	0.015 (0.003–0.072), < 0.001	.435	157
Transferrin saturation	0.941 (0.893–0.991), = 0.021	.071	157
Ferritin	1.001 (1.000–1.001), = 0.002	.151	157
CRP	1.034 (1.023–1.046), < 0.001	.657	163
Leucocytes	1.458 (1.236–1.719), < 0.001	.248	164
Haemoglobin	0.676 (0.549–0.831), < 0.001	.148	164
Neutrophils	1.629 (1.342–1.978), < 0.001	.362	164
Lymphocytes	0.029 (0.007–0.129), < 0.001	.345	164
D-Dimer	2.548 (1.542–4.210), < 0.001	.407	112
NT-pro-BNP	1.001 (1.001–1.002), < 0.001	.451	143
CD25	1.002 (1.001–1.003), = 0.001	.330	69
IL-6	1.002 (1.000–1.005), 0.064	.091	84

Table shows odds ratio (OR) of need for high-flow nasal oxygen therapy or mechanical ventilation, calculated using univariate binary logistic regression. Nagelkerke *R* square reflects the effect size of each variable. Not all variables were determined in outpatients, hence the number of patients analysed was noted (N/164). SOFA score, maximum SOFA score, Horowitz index, Troponin-T and sRAGE Ln have the largest effect size. *BMI* body mass index, *A. Hypertension* arterial hypertension, *CAD* coronary artery disease, *COPD* chronic obstructive pulmonary disease, *ACE-I* angiotensin converting enzyme inhibitor, *ARB* angiotensin II receptor blocker, *SOFA* sequential organ failure assessment score, *qSOFA* quick sequential organ failure assessment score, *sRAGE Ln* natural log transformed soluble receptor of advanced glycation end product, *AST* aspartate aminotransferase, *CRP* C-reactive protein, *NT-pro-BNP* N-terminal pro b-type Natriuretic Peptide, *CD25* soluble interleukin (IL)-2Rα, *IL-6* interleukin-6

**Table 3:** Multivariate logistic regression analysis using sRAGE to distinguish HFNO/MV and predict MV

Covariate	HFNO/MV (*n* = 32/164)		MV (*N* = 19/164)	
	OR (95% CI)	*p* value	OR (95% CI)	*p* value
sRAGE*	1.782 (1.163–2.730)	0.008	1.389 (1.060–1.820)	0.017
SOFA Score ≥ 3	106.295 (17.102–660.653)	< 0.001	66.451 (7.343–601.396)	< 0.001
Age	1.031 (0.957–1.110)	0.419		
Comorbidity	1.228 (0.161–9.397)	0.843		
Male gender	4.687 (0.568–38.704)	0.151		

^*^per 1000 pg/mL

*HFNO/MV* high-flow nasal oxygen therapy or mechanical ventilation, *sRAGE* soluble receptor of advanced glycation end product, *SOFA* sequential organ failure assessment score

### sRAGE and the need for MV

The median number of days between sRAGE measurement and the need for MV was 1 (0–4) day. sRAGE level increased not only with the maximum oxygen and ventilation therapy needed, *p* < 0.001, *η*^2^ = 0.432 (Fig. 2f) but also with ARDS severity, *p* < 0.001, *η*^2^ = 0.391 (Fig. 2g). There was a significant, moderately strong negative correlation between sRAGE and the Horowitz Index, *r*_s_ (85) = − 0.474, *p* < 0.001 (Fig. 2h).

**Fig. 3:**
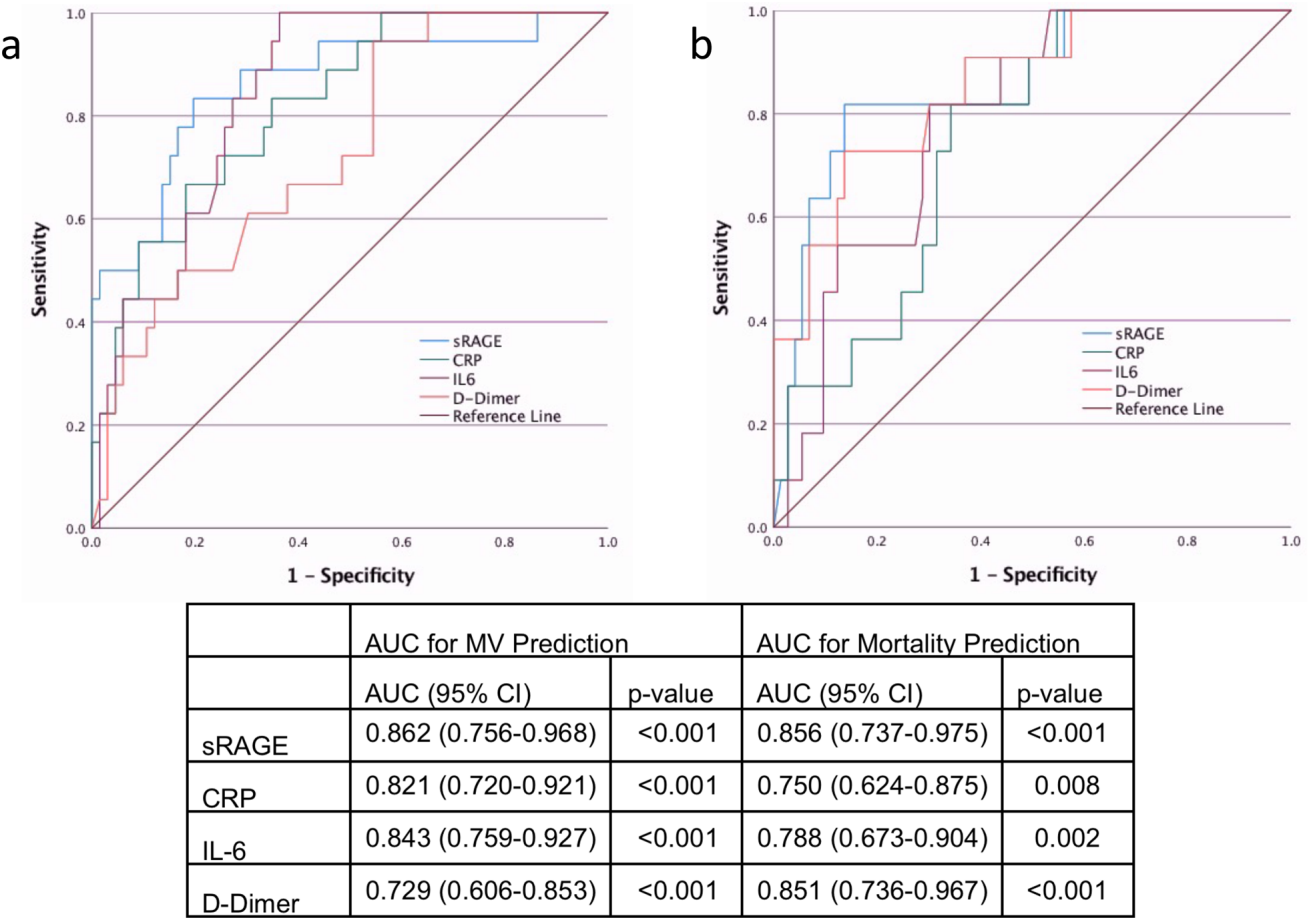
Comparison of ROC curves for predicting mechanical ventilation and mortality. Top: ROC curves for sRAGE, CRP, IL-6 and D-Dimer predicting MV for inpatients (**a**) and mortality (**b**). Bottom: AUC with 95% CI and *p* value for all variables predicting different outcomes. sRAGE is the best parameter at predicting MV and mortality. AUC: Area under the curve. ROC: receiver operating curve. sRAGE: soluble receptor for advanced glycation end products. CRP: C-reactive protein. IL-6: Interleukin-6. CI: confidence interval. MV: mechanical ventilation

When investigating the predictive value of sRAGE as a predictor of the need of MV for inpatients, the AUC in the ROC curve showed good discrimination with an AUC of 0.871 (0.770–0.973) and predicts with 84.2% sensitivity and 88.3% specificity when set at a threshold of 3108 pg/mL. Out of the seven patients that were intubated at least 2 days after the time of sampling, six out of seven (85.7%) of the patients had sRAGE levels above the threshold of 3108 pg/mL. Notably, when predicting MV only for inpatients (*N* = 84), sRAGE was the best predictor among other laboratory data when compared with CRP, IL-6 and D-Dimer (Fig. 3a). A comparison of different combinations using cut-offs of sRAGE, CRP, IL-6 and D-Dimer to predict MV in inpatients showed that the combination of high sRAGE and IL-6 had the highest AUC of 0.867 (0.762–0.972) (Additional file 3). Adjusting for SOFA Score ≥ 3, sRAGE remained associated with need for MV (Table 3). The model was able to predict MV with 95.9% sensitivity and 81.3% specificity. Adding sRAGE to the model improved the accuracy of MV prediction from 92.1 to 94.5%.

### sRAGE may help predict mortality

**Fig. 4:**
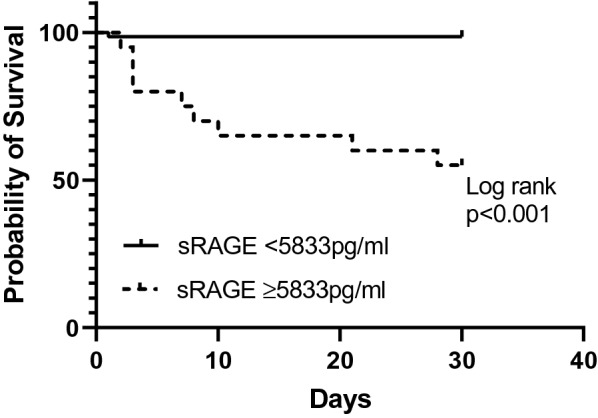
Kaplan–Meier curves in patients with sRAGE levels < 5833 pg/ml or ≥ 5833 pg/ml. Patients with sRAGE levels ≥ 5833 pg/mL showed significantly higher risk of mortality than those with sRAGE levels < 5833 pg/mL with a hazard ratio of 38.68 (8.33–179.7), *p* < 0.001. sRAGE: soluble receptor for advanced glycation end products

The median number of days between sRAGE measurement and mortality were 3 (2–10) days. When investigating sRAGE levels according to 30-day mortality, sRAGE levels were found to be much higher in patients who died [8.96 (8.68–9.49) vs 7.360 (7.015–7.825), *p* < 0.001, *η*^2^ = 0.121] (Fig. 2i). When only taking into account the cohort with the need for HFNO/MV, sRAGE levels were higher in patients who died but the difference was not statistically significant, *p* = 0.092 (Additional file 2e). The AUC in the ROC analysis of sRAGE predicting 30-day mortality was excellent, 0.903 (0.817–0.990). Setting the sRAGE threshold at 5833.13 pg/mL, 30-day mortality was correctly predicted with 81.8% sensitivity and 92.8% specificity. When predicting mortality for inpatients (*N* = 84), sRAGE was the best predictor of mortality when compared with CRP, IL-6 and D-Dimer (Fig. 3b). The corresponding Kaplan–Meier survival plots for patients with high (≥ 5833 pg/mL) v*ersus* low (< 5833 pg/mL) sRAGE levels is given in Fig. 4. In univariate Cox regression, high sRAGE (≥ 5833 pg/mL) was associated with higher risk of 30-day mortality (HR 38.68, 95% CI 8.33–179.7, *p* < 0.001).

### sRAGE increase might not be COVID-19 specific

Interestingly, there was no difference in sRAGE levels in COVID-19 compared to non-COVID pneumonia. Taking this comparison a step further by stratifying according to disease severity, only the effect of disease severity, *p* < 0.0001 but not the type of pneumonia, *p* > 0.05, or the interaction effect was significant, *p* > 0.05 (Fig. 2j). In addition, there was no difference in sRAGE levels of patients with mild COVID-19 and the healthy controls.

## Discussion

The key results of this study are as follows: sRAGE levels increased with COVID-19 severity and were found to be helpful in predicting respiratory failure with the need for MV as well as mortality from COVID-19. Interestingly, sRAGE levels in patients with COVID-19 pneumonia and non-COVID-19 pneumonia did not differ at a significant level.

Although sRAGE level increase in influenza A pneumonia (in the bronchoalveolar lavage fluid of mice) [28], community acquired pneumonia [29], sepsis [30] and lung injury are well documented [12, 31–34], there is currently limited information about how sRAGE levels vary in COVID-19. Spadaro et. al. described plasma RAGE levels in COVID-19 patients after MV [35]. To the best of our knowledge, the present study is the first to show that serum sRAGE levels increase with COVID-19 severity, measured in terms of need for hospitalisation, need for HFNO/MV, need for organ support, SOFA scores, maximum ARDS severity, and 30-day mortality. The positive correlation of sRAGE levels and duration of hospitalisation appears in line with results shown by Calfee and colleagues for lung transplantation studies [36]. In our results, sRAGE levels were 38% lower in the same patients during convalescence compared to during acute disease. This further shows that sRAGE is increased in acute COVID-19, which agrees with the findings of Dozio et al. [37] but contradicts the finding that sRAGE levels were lower in elderly patients with COVID-19 with lung involvement than in healthy controls [38]. As sRAGE levels are found to vary differently in different diseases, this finding helps to clarify serum sRAGE levels in the context of COVID-19 and adds to the existing knowledge of sRAGE.

It is known that RAGE expression is enhanced in the lung [6], specifically on epithelial alveolar type I [7] and type II lung epithelial cells [8]. Our data echo this by showing that sRAGE was negatively correlated to Horowitz Index. Beyond that, sRAGE increased as the oxygen therapy demand and ARDS severity increased. Based on these, it could be inferred that sRAGE may provide insights into the extent of lung tissue damage and could, therefore, be a valuable biomarker in COVID-19. Monitoring sRAGE levels could be useful in both outpatient and inpatient settings to support clinical decision-making, including need of hospitalisation as well as admission into the ICU.

Early detection of the potential need of MV and mortality is essential in optimising strategies used for patient monitoring and patient management. Despite efforts to optimise therapy for ARDS, mortality rates of ARDS remain high [39]. This has spurred the switch of focus in management toward primary, secondary and tertiary ARDS prevention to positively influencing patient outcomes [40]. Here, we show that a sRAGE level set at a threshold of 2348 pg/mL distinguishes COVID-19 patients with a need for HFNO/MV. This corresponds well to the median sRAGE levels reported for community acquired pneumonia without ARDS [1829.75 (1079–2629) pg/mL] and with ARDS [3296 (2168–3793) pg/mL] [29] but is higher than the sRAGE cut-off value of 1340 pg/mL for ARDS of all causes [13]. Furthermore, our results show that sRAGE level set at a threshold of 3108 pg/mL is a good predictor of the need for MV in COVID-19 inpatients, with 84.2% sensitivity and 88.3% specificity. As COVID-19 is known to cause diffuse lung damage [41], this threshold corresponds well to sRAGE cut-off values described in some [33, 42] but not in other studies [43, 44], particularly when aiming to differentiate focal and nonfocal lung damage in ARDS. This shows that measuring sRAGE levels in serum early is valuable in predicting ARDS and the need for MV and, therefore, has the potential to improve daily clinical practice.

sRAGE levels were found to be significantly higher in patients with 30-day mortality when taking into account patients of all disease severity but not significantly higher when taking into account only patients with severe disease. Interestingly, Spadaro et al. also showed, when investigating COVID-19 ARDS patients, that RAGE levels one day after MV did not differ between survivors and non-survivors [35]. Severe COVID-19 disease progression is characterised not only by lung injury but also by multiorgan failure. Therefore, monitoring sRAGE levels early may help predict mortality in the whole population by early identification of patients with severe disease, but appears to be less helpful, for patients already having severe disease. By setting the sRAGE threshold at 5833.13 pg/mL, 30-day mortality was predicted with 81.8% sensitivity and 92.8% specificity. The high sensitivity and specificity of sRAGE in predicting mortality may justify its utility in clinical practice to improve medical decisions and resource management. Jabaudon and colleagues showed that sRAGE was able to predict ARDS of different causes and ARDS mortality [13]. Our results suggest that sRAGE monitoring may also be useful in the context of COVID-19.

Several studies indicate that combining different biomarkers can be useful to improve patient management [45, 46]. Our results resonate with these findings in that a combination of sRAGE and IL-6 measurement improved the prediction of the need for MV in inpatients as compared to sRAGE, IL-6, CRP or D-Dimer.

The role of clinical and severity scores for risk-stratification in COVID-19 have been studied. SOFA score was found to be superior to qSOFA score in predicting mortality in severe COVID-19 [26]. A-DROP was found to be reliable in predicting in-hospital death in COVID-19 [47]. However, subjective dyspnea scores were found to be inadequate in assessing hypoxemia in COVID-19 [48, 49]. Interestingly, high sRAGE discriminated the need for HFNO/MV and MV independently from a high SOFA score in our multivariate models. This shows that knowledge of serum sRAGE levels brings additional value in HFNO/MV prediction.

Our study contributes to the growing body of evidence that sRAGE monitoring is clinically useful for managing patients with potential need of intensive care and MV. Measurement of sRAGE levels may assist in the selection of COVID-19 patients to be admitted into the ICU as well as their timely intubation with lung-protective ventilation. However, the cost may be a factor limiting widespread use in daily clinical practice.

In experiments with mice, RAGE-deficient mice have been shown to increase mortality in *Klebsiella pneumoniae* pneumonia but better survival in influenza A pneumonia, pneumococcal pneumonia [50] and RSV pneumonia [51]. In mice models, administration of sRAGE has also shown promising results in reducing acute lung injury [52]. Further investigations regarding RAGE expression and the effects of RAGE deficiency in COVID-19 will be required to understand whether RAGE modulation is of therapeutic value in COVID-19 patients.

It has been postulated, that RAGE plays a role in the SARS-CoV-2-mediated inflammatory response in the lungs [14, 15]. Here, it has been hypothesised, that RAGE activation is triggered by activation of the ACE/Ang II/AT1R pathway after binding of SARS-CoV-2 to Angiotensin-converting enzyme 2 (ACE2) via its spike protein (S-protein) to invade host cells. Angiotensin II increase was also hypothesised as the cause of the complex clinical picture of COVID-19 [53]. However, our data showed no difference in sRAGE levels when comparing COVID-19 and non-COVID-19 pneumonia. Furthermore, there was no difference in sRAGE levels between COVID-19 patients with mild disease and healthy controls. It could, therefore, be postulated that RAGE and Angiotensin II increase is not COVID-19 specific, but rather mirrors the severity of lung injury.

It is important to recognise the study limitations. First, the group of non-COVID-19 pneumonia cohort is small (*n* = 23) compared to the group of COVID-19 pneumonia cohort (*n* = 164) and the patients are not matched. Therefore, individual characteristics of studied patients affecting the results cannot be excluded. Further studies with more patients will contribute to the further validation of these results. Second, the bedside assessment and mechanics of breathing was not captured in this study. These factors also play a role in recognition of patients needing HFNO/MV. Third, the HFNO/MV and MV cohort as well as the number of patients with 30-day mortality was low, such that the different clinical and laboratory aspects could not be included in the regression models for more thorough interpretation of the role of sRAGE with respect to these outcomes. Therefore, continued monitoring and the inclusion of larger numbers of patients in future studies would be useful. Other limitations of this study are its retrospective analysis and single-center design as well as the lack of external validation. Furthermore, the lack of sRAGE kinetics needs to be acknowledged. It should also be noted that CT imaging of lungs was carried out only at the discretion of the attending physician and not in all patients. Therefore, future studies exploring the association of sRAGE levels with lung CT findings in COVID-19 may reveal further insights useful for improving patient care.

## Conclusions

This is to our knowledge the first study to describe elevated serum sRAGE concentrations in hospitalised COVID-19 patients, linking high sRAGE with the need of HFNO/MV, MV and 30-day mortality. Our results indicate that close monitoring of serum sRAGE levels may help to improve management of patients in the ICU and warrant further studies on RAGE modulation as a potential therapy in COVID-19.

## Supplementary Information

**Additional file 1: Table S1.** Basic characteristics of patients with COVID-19 severe disease.

**Additional file 2.** sRAGE levels vary with disease severity. sRAGE values were subject to natural log transformation (sRAGE Ln) due to highly skewed distribution. sRAGE increases as qSOFA score increases. The increase in sRAGE level is significant when qSOFA score increases from 0 to 1, *p*<0.001 and from 0 to 2, *p*<0.01 (a). There is a moderately strong significant correlation between sRAGE Ln and duration of hospitalisation, *r*_s_ (89)=0.375, *p*<0.001 (b). sRAGE Ln was significantly higher in patients with elevated D-Dimer >0.5 mg/L [U(*N*_low D-Dimer_=35, *N*_high D-Dimer_=77)=2120, *z*=4.85, *p*<0.001] (c). A Wilcoxon signed-ranks test indicated that sRAGE during convalescence was statistically significantly lower than sRAGE during acute disease, Z= -0.350, *p*<0.001 (d). Among patients with severe disease, sRAGE Ln was higher in non-survivors but the difference was not significant, [*t*(30)=-1.74, *p*=0.092] (e).

**Additional file 3.** Comparison of ROC curves using cut-offs for predicting mechanical ventilation. Top: ROC curves for sRAGE, CRP, IL-6, D-Dimer and in their combination with sRAGE using cut-offs predicting MV for inpatients. Bottom: AUC with 95% CI and *p* value for all variables predicting MV for inpatients. A combination of sRAGE and IL-6 is the best parameter at predicting MV. AUC: Area under the curve. ROC: receiver operating curve. sRAGE: soluble receptor for advanced glycation end products. CRP: C-reactive protein. IL-6: Interleukin-6. CI: confidence interval. MV: mechanical ventilation.

## Data Availability

The datasets used and/or analysed during the current study are available from the corresponding author on reasonable request.

## Abbreviations

ACE/Ang II/AT1R pathway: Angiotensin-converting enzyme/ angiotensin II/ angiotensin type 1 receptor pathway
ANOVA: Analysis of variance
ARDS: Acute respiratory distress syndrome
AUC: Area under the curve
COVID-19: Coronavirus disease 2019
ELISA: Enzyme-linked immunosorbent assay
FiO_2_: Inspired fraction of oxygen
HFNO: High-flow nasal oxygen therapy
MV: Mechanical ventilation
PaO_2_: Arterial partial pressure of oxygen
qSOFA score: Quick sequential organ failure assessment score
RAGE: Receptor of advanced glycation endproducts
ROC analysis: Receiver operating characteristic analysis
RT-qPCR: Reverse-transcriptase quantitative polymerase chain reaction
SARS-CoV-2: Severe acute respiratory syndrome coronavirus 2
SOFA score: Sequential organ failure assessment score
SpO_2_: Peripheral blood oxygen saturation
sRAGE: Soluble receptor of advanced glycation endproducts
sRAGE Ln: Natural log transformed sRAGE
