# Supplementary material for: Soluble receptor for advanced glycation end products (sRAGE) as a biomarker of COVID-19 disease severity and indicator of the need for mechanical ventilation, ARDS and mortality
Source: Ann Intensive Care. 2021 Mar 22;11:50. doi: 10.1186/s13613-021-00836-2 (PMC7983090; doi:10.1186/s13613-021-00836-2)
Supplement: Supplementary file 1 — Additional file 1: Table S1. Basic characteristics of patients with COVID-19 severe disease. [file 13613_2021_836_MOESM1_ESM.doc]

**Table S1: Basic characteristics of patients with COVID-19 severe disease.**

|  | Severe COVID-19 Pneumonia (n=32) | | |
| --- | --- | --- | --- |
|  | HFNO (n=13) | MV (n=19) | Total (n=32) |
| Gender |  |  |  |
| Male | 10 (76.9%) | 16 (84.2%) | 26(81.3%) |
| Female | 3 (23.1%) | 3 (15.8%) | 6 (18.8%) |
| Age | 68 (63-85) | 74 (63-77) | 69 (64-77) |
| BMI | 25.6 (23.8-30.4) | 26.2 (24.2-27.4) | 26.0 (24.8-26.9) |
| A.Hypertension | 9 (69.2%) | 14 (73.7%) | 23 (71.9%) |
| CAD | 2 (15.4%) | 8 (42.1%) | 10 (31.3%) |
| Diabetes Mellitus | 1 (7.7%) | 7 (36.8%) | 8 (25.0%) |
| Hyperlipidemia | 5 (38.5%) | 7 (36.8%) | 12 (37.5%) |
| Renal Insufficiency | 2 (15.4%) | 6 (31.6%) | 8 (25.0%) |
| COPD | 1 (7.7%) | 3 (15.8%) | 4 (12.5%) |
| Inflammatory Diseases | 0 (0%) | 2 (10.5%) | 2 (6.3%) |
| Malignancy | 2 (15.4%) | 3 (15.8%) | 5 (15.6%) |
| ACE-I | 6 (46.2%) | 6 (31.6%) | 12 (37.5%) |
| ARB | 1 (7.7%) | 5 (26.3%) | 6 (18.8%) |
| Cortisone | 1 (7.7%) | 5 (26.3%) | 6 (18.8%) |
| Statins | 5 (38.5%) | 6 (31.6%) | 11 (34.4%) |
| ASS/Clopidogrel | 2 (15.4%) | 7 (36.8%) | 9 (28.1%) |
| PaO2:FiO2 | 153 (135-263) | 110 (82-155) | 138 (106-179) |
| 30-day Mortality | 4 (30.8%) | 7 (36.8%) | 11 (34.4%) |
| Dialysis | 0 (0%) | 8 (42.1%) | 8 (25.0%) |
| Catecholamine | 0 (0%) | 13 (68.4%) | 13 (40.6%) |
| SOFA | 3 (2-4) | 7 (6-11) | 5 (4-7) |
| Maximum SOFA | 4 (4-6) | 13 (13-15) | 10 (5-13) |
| qSOFA |  |  |  |
| 0 | 5 (38.5%) | 4 (21.1%) | 9 (28.1%) |
| 1 | 6 (46.2%) | 11 (57.9%) | 17(53.1%) |
| 2 | 2 (15.4%) | 4 (21.1%) | 6 (18.8%) |
| 3 | 0 (0%) | 0 (0%) | 0 (0%) |

Table shows the demographic characteristics, medical history and clinical data of subset of cohort with severe COVID-19 pneumonia with need for high-flow nasal oxygen therapy (HFNO) and mechanical ventilation (MV). Nominal and ordinal variables are reported as count (percentage in cohort), continuous variables are reported as median (IQR). BMI: body mass index; A. Hypertension: arterial hypertension; CAD: coronary artery disease; COPD: chronic obstructive pulmonary disease; ACE-I: angiotensin converting enzyme inhibitor; ARB: angiotensin II receptor blocker; SOFA: sequential organ failure assessment score; qSOFA: quick sequential organ failure assessment score.
