# Supplementary figures and images for: Soluble receptor for advanced glycation end products (sRAGE) as a biomarker of COVID-19 disease severity and indicator of the need for mechanical ventilation, ARDS and mortality
Source: Ann Intensive Care. 2021 Mar 22;11:50. doi: 10.1186/s13613-021-00836-2 (PMC7983090; doi:10.1186/s13613-021-00836-2)

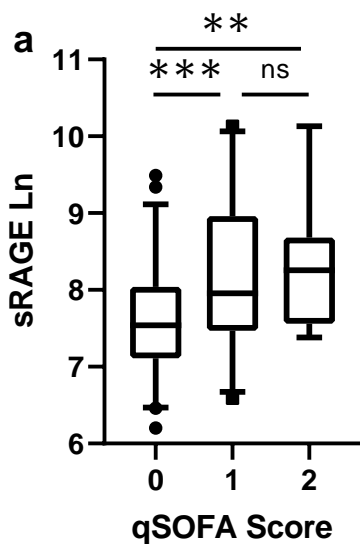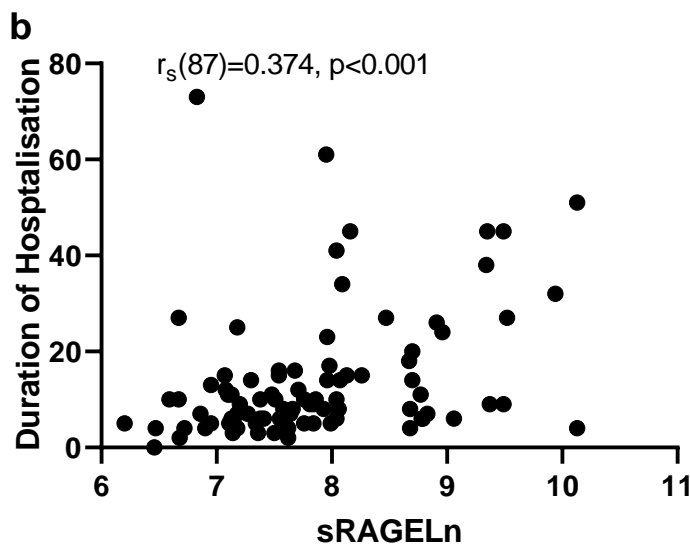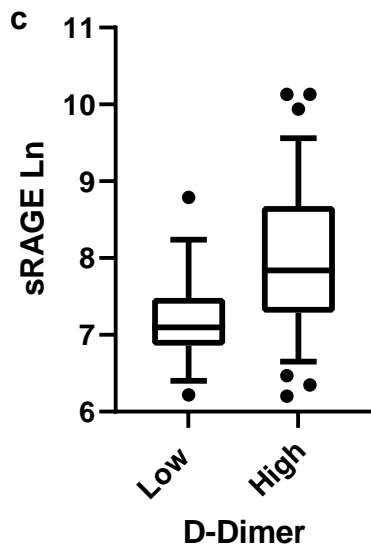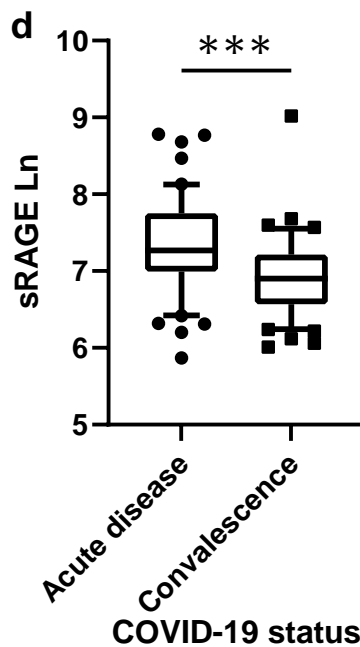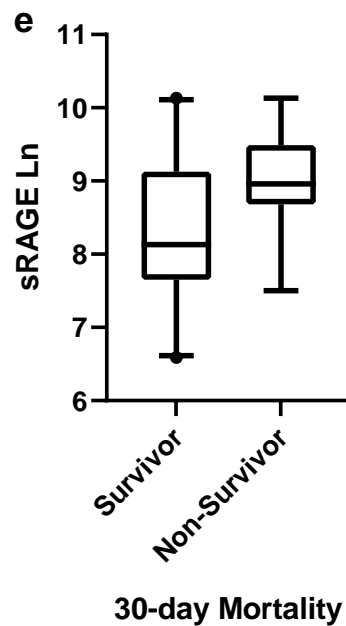

Supplement: Supplementary file 2 — Additional file 2. sRAGE levels vary with disease severity. sRAGE values were subject to natural log transformation (sRAGE Ln) due to highly skewed distribution. sRAGE increases as qSOFA score increases. The increase in sRAGE level is significant when qSOFA score increases from 0 to 1, p<0.001 and from 0 to 2, p<0.01 (a). There is a moderately strong significant correlation between sRAGE Ln and duration of hospitalisation, rs (89)=0.375, p<0.001 (b). sRAGE Ln was significantly higher in patients with elevated D-Dimer >0.5 mg/L [U(Nlow D-Dimer=35, Nhigh D-Dimer=77)=2120, z=4.85, p<0.001] (c). A Wilcoxon signed-ranks test indicated that sRAGE during convalescence was statistically significantly lower than sRAGE during acute disease, Z= -0.350, p<0.001 (d). Among patients with severe disease, sRAGE Ln was higher in non-survivors but the difference was not significant, [t(30)=-1.74, p=0.092] (e). [file 13613_2021_836_MOESM2_ESM.pdf]

## Slide 1
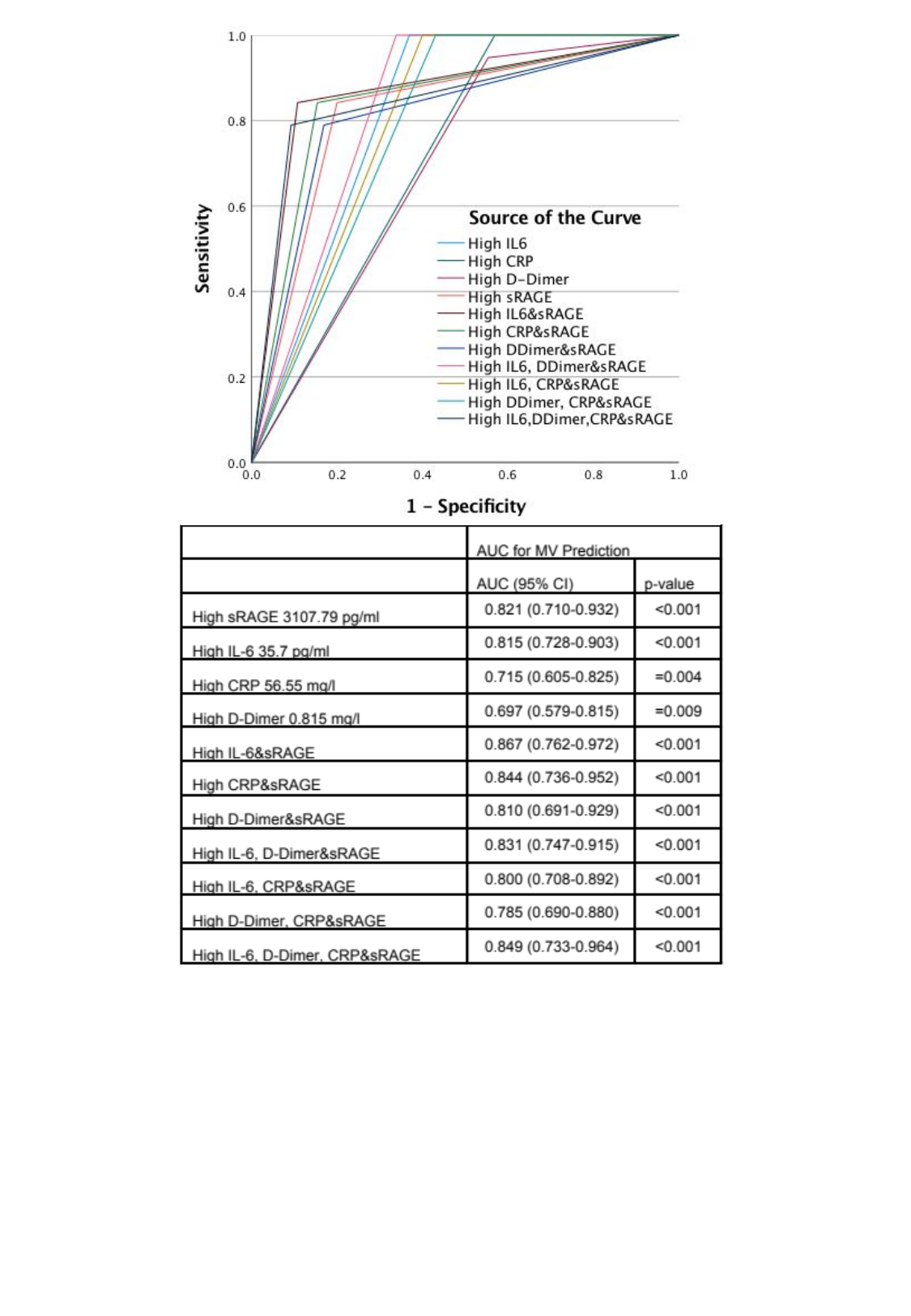

Supplement: Supplementary file 3 — Additional file 3. Comparison of ROC curves using cut-offs for predicting mechanical ventilation. Top: ROC curves for sRAGE, CRP, IL-6, D-Dimer and in their combination with sRAGE using cut-offs predicting MV for inpatients. Bottom: AUC with 95% CI and p value for all variables predicting MV for inpatients. A combination of sRAGE and IL-6 is the best parameter at predicting MV. AUC: Area under the curve. ROC: receiver operating curve. sRAGE: soluble receptor for advanced glycation end products. CRP: C-reactive protein. IL-6: Interleukin-6. CI: confidence interval. MV: mechanical ventilation. [file 13613_2021_836_MOESM3_ESM.ppt]
